# Leisure-time physical activity trajectories from adolescence to adulthood in relation to several activity domains: a 27-year longitudinal study

**DOI:** 10.1186/s12966-023-01430-4

**Published:** 2023-03-09

**Authors:** Frida Kathrine Sofie Mathisen, Torbjørn Torsheim, Coral Falco, Bente Wold

**Affiliations:** 1 Department of Health Promotion and Development, University of Bergen, Bergen, Norway; 2 Department of Psychosocial Science, University of Bergen, Bergen, Norway; 3 Department of Sport, Food and Natural Sciences, Western Norway University of Applied Sciences, Bergen, Norway

**Keywords:** Physical activity, Activity domains, Organised sports, Outdoor recreation, Diversity in leisure-time activities, Peers, Latent class growth analysis, Longitudinal, Adolescence, Adulthood

## Abstract

**Background:**

Insufficient physical activity (PA) levels among adolescents and adults make promoting PA a public health priority. Although most people exhibit low or decreasing levels of PA, other groups increase or maintain high levels of activity. These different groups may engage differently in activity domains during their leisure time. This study aimed to identify distinct trajectories of leisure-time vigorous physical activity (LVPA) and to explore whether these trajectories are characterised by differences in four activity domains (participation in organised sports clubs, diversity in leisure-time activities, outdoor recreation, and peer PA) over the life course.

**Methods:**

Data were drawn from the Norwegian Longitudinal Health Behaviour Study. The sample of participants (*n* = 1103, 45.5% female) was surveyed 10 times from age 13 years in 1990 to age 40 years in 2017. LVPA trajectories were identified using latent class growth analysis, and mean differences in activity domains were studied using the one-step BCH approach.

**Results:**

Four trajectories were identified: active (9%), increasingly active (12%), decreasingly active (25%), and low active (54%). Overall, this analysis showed a declining tendency in LVPA from age 13 to 40 years except for the increasingly active trajectory. Belonging to a trajectory with a higher LVPA level was related to higher mean levels of the included activity domains. Compared with those in the increasing trajectory, people belonging to the decreasing trajectory reported higher mean participation levels in and age at becoming a member of sports clubs, diversity in leisure-time activities, and best friend’s activity level during adolescence. However, in young adulthood, people in the increasingly active trajectory reported significantly higher mean levels for the same variables.

**Conclusions:**

The development of LVPA from adolescence to adulthood is heterogeneous, suggesting the need for targeted health promotion initiatives. The largest trajectory group included more than 50 percent and was characterized by low levels of LVPA, less engagement in PA domains and fewer active friends. There seems to be little carry-over effect of engagement in organised sports in adolescence regarding level of LVPA later in life. Changes in social surroundings throughout the life span, such as having friends who are more or less engaged in PA, may assist or hinder health enhancing engagement in LVPA.

**Supplementary Information:**

The online version contains supplementary material available at 10.1186/s12966-023-01430-4.

## Background

Physical activity (PA) is well established as a predictor of lifetime health and is essential to the inclusion of health promotion in global health policies and local interventions. Nevertheless, global estimates show that 27.5% of adults [1] and 81% of adolescents [2] do not meet the global recommendations for aerobic exercise, which is at least 150–300 min of moderate-to-vigorous intensity, or at least 75–150 min of vigorous intensity, or a combination of those throughout the week [3]. As pointed out by the recommendations, there seems to be an additional health benefit related to vigorous physical activity, and it has been shown that for the same amount of total PA, higher proportions of PA with vigorous intensity are related to lower mortality [4]. Research related to the mechanisms that promote lifelong PA and decrease inactivity is needed, especially research based on longitudinal data. Longitudinal data allow researchers to examine the life-course patterns of PA over time and across various life events and transitions [5].

To understand further these changes over time and differences between individuals, four approaches have been suggested by Telama [6]: the carry-over value hypothesis, ability and readiness hypothesis, habit-formation hypothesis, and self-selection hypothesis. The carry-over value hypothesis suggests that adults continue to engage in activities they participated in at a young age. The ability and readiness hypothesis suggests that earlier experience and the basic skills connected to this experience contribute to the maintenance of or re-engagement in PA despite participation in different types of activity. The habit-formation hypothesis suggests that behaviour is repeated because it is a habit and that this behaviour is based not only on planned behaviour but is automatic and performed with less awareness. The self-selection hypothesis acknowledges a hereditary disposition to fitness and motor performance in some people, which makes them engage in PA more often in adolescence and adulthood. These hypotheses can be used individually to explain the possible paths of lifelong patterns of PA or they can be applied in a cumulative way through the combination of more than one hypothesis to explain the establishment of an active lifestyle.

A growing body of research has used finite mixture modelling to investigate the development of PA from a life-course perspective. The literature was recently summarised in a systematic review [7]. The number of previous studies looking at the development of PA from childhood or adolescence to adulthood is limited, and only four studies, based on only two different data materials, both population-based studies from Finland, were identified in the systematic review. These studies identified three or five latent leisure-time PA trajectories and that the most significant proportion of people follow a stable moderate or persistently low level of PA. One study identified two additional trajectories in addition to the three prevalent trajectories (steady high, moderate, or low level of leisure-time PA) that comprised an increasingly active or a decreasingly active trajectory [8].

Emerging research interest in the domains related to PA (i.e., the context in which PA occurs) has also contributed to the knowledge about lifelong engagement in leisure-time PA [9]. Diverse patterns of engagement in PA, both organised and non-organised, in team sports or individual sports, and in a wide variety of activities during adolescence are related to the activity level in adulthood (e.g., [10–15]).

Organised sport provides structures for social interaction and skill development, which are thought to contribute to the development of lifelong PA by establishing habits, abilities, and continued participation. A previous study [16] indicated that participation in sports clubs is associated with a sustained or increased PA pattern and, correspondingly, drop out from organised sport with a decrease in PA from a high level. Kjønniksen and colleagues [12] found that diversity in leisure-time activities (the number of activities participated in) at age 15 years was more strongly related to later activity level at age 23 years than was engagement in specific activities. Earlier experiences with PA and sports make it easy to maintain or re-engage in PA, especially if the newer form of PA differs from the earlier activity [6]. Previous research also indicates that broad and varied experiences during adolescence affect PA habits later in life [11], a finding that is consistent with the ability and readiness hypothesis [6].

Another domain related to PA, and possibly relevant to the Nordic context, is the concept of nature-related outdoor recreation, *friluftsliv*, which is considered a core social and cultural value in Norway [17]. Different forms of outdoor activities may support PA throughout the entire life course. Participation in outdoor PA influences positive attitudes towards PA and contributes to positive activity habits [18]. PA during childhood and adolescence is usually performed with peers, and peers appear to influence the individual’s PA level significantly through behavioural modelling, peer pressure, group norms, and co-participation [19]. When entering adulthood, friends and partners may act as critical agents for activity and inactivity.

There is an evidence gap in the research literature needed to develop a more nuanced understanding of PA development from adolescence into adulthood [7, 9]. This study aimed to identify distinct trajectories of leisure-time vigorous physical activity (LVPA) and to explore whether these trajectories are characterised by differences related to activity domains over the life course. The activity domains included were participation in organised sports, diversity in leisure-time activities, outdoor recreation, and active peers.

## Methods

### Study sample

Data were drawn from the Norwegian Longitudinal Health Behaviour Study. The study involved participants from 22 randomly selected schools in Hordaland in Western Norway. The sample was geographically limited to this region, which allowed the researchers to maintain close contact with the participants at the beginning of the project to establish a good foundation for this cohort study. A total of 924 students (414 girls, 44.8%) participated in the first survey in 1990. This was 78% of the initial sample of 1195 students, and the average age was 13.3 years. During the two subsequent data collections in school, any new student in any randomly selected school was invited to participate. This meant that a total of 1105 people participated in the survey at least once over the 27 years (89% of the total invited sample of 1242). Written consent was given by parents before participation in the survey. Participants were surveyed 10 times (1990, 1991, 1992, 1993, 1995, 1996, 1998, 2000, 2007, and 2017). For the first three times, the survey was conducted during school hours and the students completed the self-completed questionnaires in class. After that, the questionnaire was distributed by post. Participants were also given an option to respond online for the last two surveys. The questionnaire was distributed during October, with greater variation in time of completion when the survey was sent by post. More information about the sample is found in Additional file 1.

Of the total sample of 1105 participants, only participants having at least one measure of LVPA over the 10 measurement points were included in the analyses, which gave a sample of 1103 participants (45.5% female).

#### Outcome measure

The outcome measure was LVPA. To assess the participants’ level of LVPA, a previously used item from The Health Behaviour in School-aged Children (HBSC) Study: WHO Collaborative Cross-national Study was included in the questionnaire [20]. The question reads, “Outside school hours, how often do you do sports or exercise to the extent that you become out of breath or sweat?” The following response categories are offered (coding in parenthesis): Every day (7), 4–6 times a week (5), 2–3 times a week (2.5), Once a week (1), Once a month (0.25), Less than once a month (0), and Never (0). In 1993, the first part of the question was changed to “Outside school hours/work”. This question was included at all 10 measurement points and has previously been identified as having acceptable to good reliability in an Australian sample [21] and overall good reliability in a Norwegian sample aged 13–18 years [22]. Validity has been found to be fair when correlated with maximal oxygen uptake, especially among girls [22].

#### Auxiliary variables

##### Membership in sports clubs

The participants’ membership status in organised sports was assessed using the question, “Are you a member of a sports club or sports association?” The following response categories were offered: Yes (1), No, but I have been a member before (0), and No, I have never been a member of a sports club (0). This item was included eight times and was excluded at ages 19 and 21 years.

##### Age at becoming a member of a sports club

The participants were asked retrospectively four times from 1990–1993 the question, “When did you become a member of a sports club?”. The response categories were I have never been a member of a sports club or sports association (0) and I became a member when I was about …. years old, with response options of 1–13, 14, 15 or 16 years. Data from these four measurements were recoded into one where the most initial response was preferred.

##### Diversity in leisure-time activities

A list of alternative types of sport or exercise was provided to respondents at the ages of 15 years (33 alternatives), 23 years (33 alternatives, exercise in a fitness centre was added and orienteering was removed), and 40 years (20 alternatives). See complete list of all activities in Additional file 2. Participants recorded the frequency level for all activities using four response categories: Several times a week, Once a week, Less than once a week, and Never. A count variable was computed to measure diversity in leisure-time activities. Performance of a sport or exercise at any frequency was counted in.

##### Outdoor recreation

The questions about outdoor recreational activity were, “How often do you usually do outdoor activity in summer? Outdoor recreation in summer can include hiking, swimming, cycling, or fishing” and “How often do you usually do outdoor activity in winter? Outdoor recreation in winter can include hiking, fishing or cross-country skiing”. The following response categories were offered: Four times a week or more often (4), 2–3 times a week (3), Once a week (2), Less than once a week (1), and Never (0). These items were included at ages 13, 14, 15, 16, 23, 30, and 40 years.

##### Active friends

Peer PA was assessed using two questionnaire items. The first related to the number of friends participating in sports and included the question, “How many of your friends do sports or exercise?”. The response categories were Almost all (4), More than half (3), About half (2), Less than half (1), and None (0). This item was measured at ages 13, 15, and 18 years.

The second peer item related to the level of sports and exercise performed by the participant’s best friend and read, “Does your best friend do sports and exercise?” The responses options were Four times a week or more (4), 2–3 times a week (3), Once a week (2), Less than once a week (1), Never (0), and I do not have a best friend (missing). This item was measured at ages 13, 15, and 23 years.

Gender (binary, measured at baseline), having children (yes/no, measured at ages 21, 23, 30 and 40 years), income (gross income in intervals of 100 000 NOK, measured at ages 23, 30 and 40 years), and body mass index (BMI, measured at ages 15, 23 and 40 years), which was calculated based on self-reported height and weight, were included to describe the characteristics of the different trajectory classes because previous research has found them to be related to the development of lifetime PA [7, 8, 16, 23–28].

### Statistical analysis

The data were managed, and descriptive statistics were calculated using IBM SPSS (version 27.0). The data were converted to Mplus (version 8.7 [29]) for the latent class growth analysis (LCGA). The level of statistical significance was 0.05.

Prior to the LCGA, the data on LVPA from all 10 measurement points were modelled in a latent growth model to explore what number of growth parameters suited the data best. Three models were tested, with two (intercept and slope factor), three (intercept, slope, and quadratic slope factor) and four (intercept, slope, quadratic, and cubic slope factor) growth parameters. The best fit, determined by the highest comparative fit index (CFI = 0.911) and the lowest root mean square error of approximation (RMSEA = 0.05) was obtained when the model included four growth parameters. The LCGA was then fitted using the same growth parameters.

#### Latent class growth analysis

Trajectories for LVPA were identified using LCGA, a type of group-based trajectory model, which makes it possible to identify latent classes of individuals based on their joint growth trajectories over time [30]. The LVPA variable was treated as continuous in the analysis.

Missing data were assumed to be missing at random (MAR) and addressed using full information maximum likelihood estimation (FIML). The model parameters were estimated using the maximum likelihood estimator with robust standard errors (MLR). The number of classes was determined by testing the model fit for two, three, four, five, six, and seven latent trajectory classes.

Akaike information criterion (AIC), Bayesian information criterion (BIC), entropy, average posterior probability > 0.70 for within-group membership, the Vuong–Lo–Mendell–Rubin test (VLMR), and the bootstrap likelihood ratio test (BLRT) were used to assess the fit and interpretability of the model and number of latent trajectory classes [31].

When assessing the class enumeration, we did not find that the AIC and BIC values reached a low point and started to increase. The VLMR test was significant only for the two-class and four-class models (*p* < 0.05). However, the proposed more reliable BLRT was significant for all models. The enumeration was therefore based on entropy and the posterior probability of class membership. Theoretical and empiric support for the four-class solution was also used [31]. The qualitative assessment of the three-class model and five-class model vs the four-class model supported the decision because the three-class solution provided fewer nuances and the five-class solution had two similar and (almost) parallel classes. Figures showing plots of the two- to seven-class models are presented in Additional file 3.

#### Distal outcome model using the Block–Croon–Hagenaars (BCH) approach

The mean differences in the above-mentioned variables related to activity domains (e.g., membership in sports clubs, diversity in leisure-time activity, peer PA) measured in adolescence, young adulthood, and adulthood were studied across the trajectory classes. To do this, we used the one-step automatic BCH approach. Using this approach to estimate a distal outcome model allowed us to avoid shift of the latent class trajectories so that they were no longer measured only by the LVPA indicator. Instead, the BCH method avoids a shift in the latent classes (i.e., LVPA trajectories) by using a weighted multiple group analysis in the final stage [32]. In the BCH approach, the auxiliary variables are treated as continuous.

### Quality assessment

The Guidelines for Reporting on Latent Trajectory Studies (GRoLTS) [31] checklist was used to ensure the quality of the analysis (see Additional file 4).

## Results

### Drop out analysis

Drop out analysis were undertaken by comparing baseline values of LVPA, membership in sports club, age at becoming a member of a sport club, outdoor recreation, number of active friends and best friend’s activity level to examine whether there was a difference between those who dropped out of the study before age 40 and the 455 respondents who did not. Independent sample t-tests showed no statistically significant differences between these two groups (*p* > 0.05*)*.

### Participants and descriptive statistics

Among the total sample, 17% of the participants completed all 10 repeated measurements of LVPA; 15% completed nine measurements, 11% eight, 9% seven, 10% six, 9% five, 11% four, 10% three, 5% two, and 4% one. Table 1 shows the descriptive statistics for all included variables at all 10 measurement points from 1990 to 2017.

**Table 1:** Descriptive statistics of included variables by measurement point

*Year*	1990	1991	1992	1993	1995	1996	1998	2000	2007	2017
*Respondent age*	13	14	15	16	18	19	21	23	30	40
*N*	924	958	936	789	779	643	634	630	536	455
*Coding of time in LCGA*	-10	-9	-8	-7	-5	-4	-2	0	7	17
*LVPA (0–7)*										
n	912	952	945	708	777	639	583	628	533	447
Mean	3.18	3.11	3.00	2.55	2.24	2.11	2.02	1.88	1.70	1.99
S.E	0.07	0.07	0.07	0.08	0.08	0.08	0.08	0.08	0.07	0.08
*Membership in sports clubs (No [0], Yes [1])*										
n	913	953	940	708	776			628	533	447
Percentage member	65.4	62.4	58.5	52.2	38.8			26.8	23.1	30.5
*Diversity in leisure-time activities*										
n			927					627		452
Mean			13.57					9.02		7.57
S.E			0.21					0.18		0.16
*Outdoor recreation, summer (0-4)*										
n	917	949	946	703				628	533	446
mean	3.30	3.12	3.30	2.99				2.84	2.92	3.01
S.E	0.03	0.03	0.03	0.04				0.04	0.04	0.04
*Outdoor recreation, winter (0-4)*										
n	915	949	945	704				628	534	443
mean	2.94	2.55	2.55	2.23				2.03	2.23	2.32
S.E.	0.03	0.03	0.04	0.04				0.04	0.04	0.05
*Number of active friends (0–4)*										
n	904		934		772					
Mean	3.16		2.75		2.15					
S.E	0.04		0.04		0.05					
*Best friend’s activity level (0–4)*										
n	878		934					626		
Mean	2.65		2.58					1.93		
S.E	0.04		0.04					0.05		
*Gender (Boy [0], Girl [1])*										
n^a^	1103									
Percentage of girls	45.6									
*BMI*										
n			886					606		443
Mean			20.18					23.28		25.52
S.E			0.08					0.15		0.18
*Having children (No [0], Yes [1])*										
n							585	628	536	449
Percentage having children							7.0	16.4	55.0	85.3
*Income (2000: 1–6; 2007: 1–8; 2017: 1–10)*										
n								626	534	446
Mean								1.66	3.78	6.45
S.E								0.04	0.07	0.10

The range for the measure of diversity in leisure-time activity was in 1992 0 to 33; in 2000 0–27; and in 2017 0–20. The range for the measure of BMI was in 1992 13.72–33.91; in 2000 14.69–53.98; in 2017 18.36–41.40

*LCGA* Latent class growth analysis, *LVPA* Leisure-time vigorous physical activity, *BMI* Body mass index

^a^The *n* is based on data from all measurement points

### LVPA trajectories

**Fig. 1:**
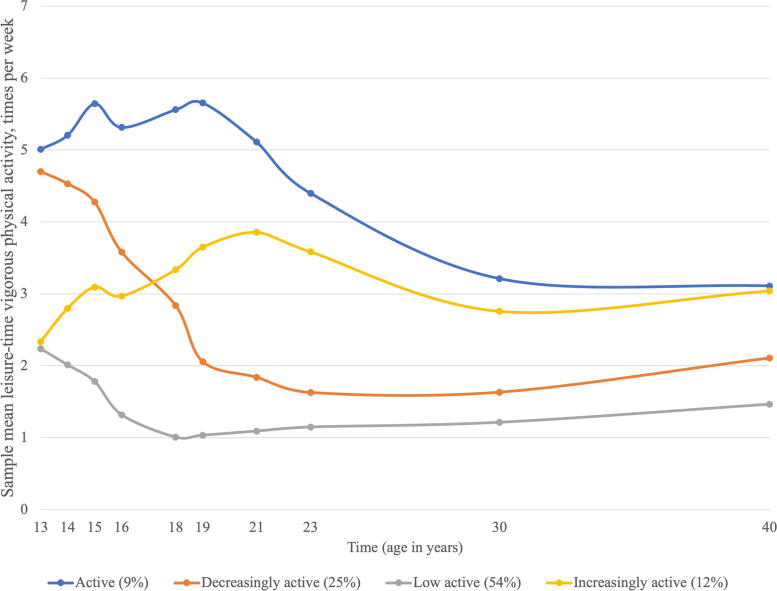
Leisure-time vigorous physical activity trajectories (*n* = 1103)

Four LVPA trajectories were identified from adolescence to adulthood (Fig. 1). Based on their development, the trajectories were named (ranked from smallest to largest and with the percentage given in parentheses): active (9%), increasingly active (12%), decreasingly active (25%), and low active (54%)*.* The final number of trajectories was selected based on the described fit statistics (Table 2).

**Table 2:** Latent Class Growth Analysis (LCGA) based on the total sample

*No. of classes*	*AIC*	*BIC*	*BLRT*	*VLMR*	*Entropy*	*Average Latent Class Probabilities for Most Likely Latent Class Membership (%)*	*Sample Size Per Class Based on Most Likely Class Membership*	*The Number of Random Start Values and Final Iterations*
1	29,901.46	29,971.54	––	––	––	100	1103	200, 20
2	28,520.17	28,615.28	*p* < .05	*p* < .05	0.773	92/94	324/779	200, 20
3	28,248.55	28,368.69	*p* < .05	*p* = .07	0.690	89/77/85	612/342/149	200, 20
**4**	**28,090.97**	**28,236.14**	***p* < .05**	***p* < .05**	**0.703**	**84/78/87/75**	**102/276/597/128**	**200, 20**
5	27,997.88	28,168.08	*p* < .05	*p* = .20	0.664	77/72/71/82/87	122/292/169/471/49	200, 20
6	27,928.51	28,123.74	*p* < .05	*p* = .61	0.664	81/85/70/71/72/86	483/46/268/121/141/44	200, 20
7	27,871.38	28,091.64	*p* < .05	*p* = .11	0.653	87/84/78/74/72/71/62	41/34/432/166/62/190/178	200, 20

*AIC* Akaike information criterion, *BIC* Bayesian information criterion, *BLRT* Bootstrap likelihood ratio test, *VLMR* Vuong-Lo-Mendell-Rubin test

The class solution considered optimal is presented in bold

The active and decreasingly active trajectories started with the highest LVPA level at the baseline. From here, the active trajectory continued to exhibit higher levels of LVPA than the three other trajectories. The decreasingly active trajectory showed a continuous decrease in activity level from adolescence to young adulthood but exhibited a more stable pattern after age 23 years. The low active trajectory had the lowest mean LVPA at age 13 years and showed a decreasing level of LVPA until the age of 18 years. From here, this trajectory continued at about the same low level until the age of 40 years. The increasing trajectory started with about the same estimated mean LVPA level as the low active trajectory. However, it ended at about the same LVPA level as the active trajectory at age 40 years and was the only trajectory in which the mean LVPA level increased from age 13 to age 40 years.

### Characteristics related to activity domains across the LVPA trajectories

**Fig. 2:**
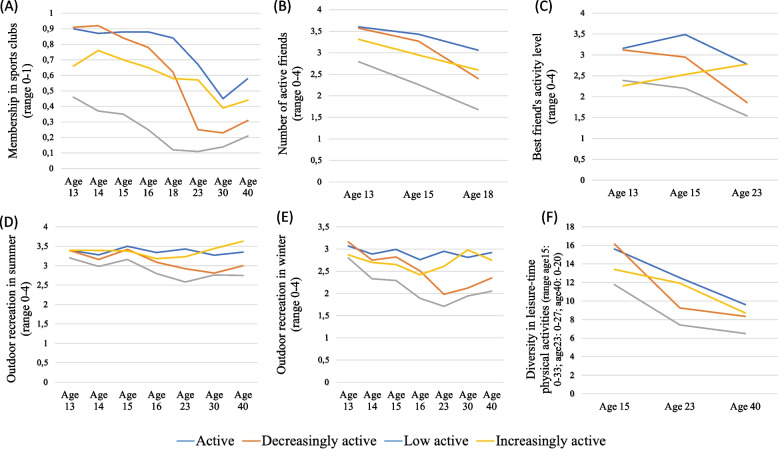
Mean values for activity domains measured multiple times (**A** = membership in organised sports clubs; **B** = number of active friends; **C** = best friend’s activity level; **D** = Outdoor recreation in summer; **E** = Outdoor recreation in winter; **F** = diversity in leisure-time activities) across the leisure-time vigorous physical activity trajectories

The mean and standard error of all included auxiliary variables across all four trajectories and indications of significant differences between each trajectory are shown in Table 3. The development within the four trajectories is illustrated in Fig. 2.

**Table 3:** Distal outcome model using the Block-Croon-Hagenaars (BCH) approach for included activity domains, demographic and socioeconomic variables

	Active^a^		Decreasingly active^b^		Low active^c^		Increasingly active^d^		*p*					
	Mean	S.E	Mean	S.E	Mean	S.E	Mean	S.E	a vs. b	a vs. c	a vs. d	b vs. c	b vs. d	c vs. d
*Membership in sports clubs*														
Age 13	0.90	0.04	0.91	0.03	0.46	0.03	0.66	0.06	0.773	** < 0.001**	**0.003**	** < 0.001**	**0.001**	**0.004**
Age 14	0.87	0.05	0.92	0.03	0.37	0.03	0.76	0.06	0.362	** < 0.001**	0.158	** < 0.001**	**0.018**	** < 0.001**
Age 15	0.88	0.05	0.84	0.04	0.35	0.03	0.70	0.06	0.540	** < 0.001**	**0.022**	** < 0.001**	0.057	** < 0.001**
Age 16	0.88	0.05	0.78	0.04	0.25	0.03	0.65	0.07	0.190	** < 0.001**	**0.012**	** < 0.001**	0.137	** < 0.001**
Age 18	0.84	0.06	0.62	0.05	0.12	0.02	0.58	0.07	**0.005**	** < 0.001**	**0.003**	** < 0.001**	0.649	** < 0.001**
Age 23	0.67	0.08	0.25	0.05	0.11	0.02	0.57	0.07	** < 0.001**	** < 0.001**	0.386	**0.026**	**0.002**	** < 0.001**
Age 30	0.45	0.10	0.23	0.05	0.14	0.03	0.39	0.07	0.061	**0.002**	0.610	0.130	0.117	**0.004**
Age 40	0.58	0.10	0.31	0.06	0.21	0.03	0.44	0.09	**0.030**	** < 0.001**	0.323	0.177	0.232	**0.016**
Age when first member	7.20	0.28	7.62	0.19	8.44	0.14	8.54	0.34	0.253	** < 0.001**	**0.004**	**0.001**	**0.029**	0.798
Number of times reported member	4.18	0.25	3.66	0.15	1.41	0.09	3.66	0.25	0.098	** < 0.001**	0.153	** < 0.001**	1.000	** < 0.001**
*Diversity in physical activities*														
Age 15	15.61	0.80	16.16	0.53	11.77	0.36	13.41	0.71	0.592	** < 0.001**	**0.049**	** < 0.001**	**0.004**	0.052
Age 23	12.52	0.65	9.26	0.49	7.42	0.28	11.92	0.56	** < 0.001**	** < 0.001**	0.508	**0.002**	**0.001**	** < 0.001**
Age 40	9.62	0.64	8.35	0.38	6.50	0.26	8.71	0.57	0.109	** < 0.001**	0.306	** < 0.001**	0.623	**0.001**
*Outdoor recreation*														
Age 13, summer	3.40	0.12	3.39	0.07	3.20	0.05	3.40	0.11	0.922	0.110	0.981	**0.040**	0.939	0.110
Age 13, winter	3.07	0.12	3.16	0.08	2.8	0.05	2.87	0.12	0.585	**0.037**	0.243	** < 0.001**	0.057	0.640
Age 14, summer	3.28	0.12	3.16	0.08	2.98	0.06	3.39	0.10	0.450	**0.020**	0.473	0.084	0.099	**0.001**
Age 14, winter	2.89	0.13	2.75	0.08	2.33	0.06	2.70	0.12	0.384	** < 0.001**	0.306	** < 0.001**	0.785	**0.006**
Age 15, summer	3.50	0.9	3.42	0.07	3.16	0.05	3.38	0.11	0.522	**0.001**	0.427	**0.008**	0.801	0.079
Age 15, winter	2.99	0.13	2.82	0.09	2.29	0.06	2.65	0.13	0.301	** < 0.001**	0.065	** < 0.001**	0.304	**0.014**
Age 16, summer	3.34	0.13	3.09	0.09	2.80	0.07	3.18	0.12	0.127	** < 0.001**	0.358	**0.016**	0.582	**0.009**
Age 16, winter	2.76	0.16	2.51	0.09	1.89	0.07	2.42	0.13	0.202	** < 0.001**	0.105	** < 0.001**	0.578	**0.001**
Age 23, summer	3.43	0.14	2.92	0.10	2.58	0.07	3.23	0.12	**0.007**	** < 0.001**	0.317	**0.008**	0.073	** < 0.001**
Age 23, winter	2.95	0.17	1.98	0.12	1.71	0.06	2.61	0.14	** < 0.001**	** < 0.001**	0.145	0.054	**0.002**	** < 0.001**
Age 30, summer	3.27	0.16	2.81	0.12	2.76	0.07	3.44	0.10	**0.027**	**0.002**	0.394	0.689	** < 0.001**	** < 0.001**
Age 30, winter	2.81	0.20	2.12	0.12	1.94	0.07	2.98	0.13	**0.006**	** < 0.001**	0.506	0.216	** < 0.001**	** < 0.001**
Age 40, summer	3.35	0.14	3.00	0.10	2.75	0.08	3.63	0.11	0.056	** < 0.001**	0.127	0.069	** < 0.001**	** < 0.001**
Age 40, winter	2.92	0.15	2.35	0.12	2.05	0.08	2.75	0.16	**0.006**	** < 0.001**	0.447	**0.047**	0.060	** < 0.001**
*Number of active friends*														
Age 13	3.60	0.10	3.57	0.07	2.79	0.07	3.31	0.14	0.796	** < 0.001**	0.099	** < 0.001**	0.117	**0.002**
Age 15	3.43	0.12	3.27	0.10	2.26	0.07	2.95	0.15	0.350	** < 0.001**	**0.015**	** < 0.001**	0.090	** < 0.001**
Age 18	3.06	0.15	2.40	0.13	1.68	0.07	2.60	0.17	**0.002**	** < 0.001**	0.053	** < 0.001**	0.382	** < 0.001**
Best friend’s activity level														
Age 13	3.16	0.13	3.12	0.09	2.39	0.06	2.26	0.14	0.848	** < 0.001**	** < 0.001**	** < 0.001**	** < 0.001**	0.434
Age 15	3.49	0.11	2.95	0.10	2.20	0.07	2.53	0.14	** < 0.001**	** < 0.001**	** < 0.001**	** < 0.001**	**0.021**	**0.048**
Age 23	2.78	0.23	1.86	0.15	1.54	0.08	2.78	0.18	**0.002**	** < 0.001**	0.990	0.071	** < 0.001**	** < 0.001**
Gender	0.22	0.05	0.30	0.04	0.56	0.02	0.55	0.06	0.206	** < 0.001**	** < 0.001**	** < 0.001**	**0.002**	0.939
*Body mass index*														
Age 15	20.11	0.31	20.16	0.18	20.18	0.14	20.29	0.27	0.903	0.849	0.686	0.952	0.729	0.733
Age 23	23.79	0.45	23.29	0.37	23.13	0.25	23.46	0.50	0.422	0.193	0.640	0.752	0.795	0.581
Age 40	24.39	0.62	25.88	0.40	25.62	0.31	25.30	0.75	0.059	0.069	0.371	0.638	0.532	0.711
*Having children*														
Age 21	0.02	0.03	0.05	0.03	0.11	0.02	0.01	0.02	0.380	**0.003**	0.947	0.098	0.330	**0.003**
Age 23	0.10	0.05	0.19	0.05	0.19	0.02	0.08	0.04	0.265	0.113	0.740	0.905	0.123	**0.037**
Age 30	0.43	0.10	0.53	0.06	0.61	0.04	0.47	0.08	0.391	0.069	0.728	0.272	0.574	0.122
Age 40	0.87	0.07	0.96	0.03	0.81	0.03	0.79	0.07	0.246	0.457	0.445	**0.002**	**0.036**	0.776
*Income*														
Age 23	1.64	0.15	1.75	0.11	1.68	0.05	1.48	0.11	0.594	0.784	0.402	0.626	0.118	0.115
Age 30	5.00	0.35	3.86	0.18	3.62	0.10	3.53	0.22	**0.008**	** < 0.001**	**0.001**	0.276	0.283	0.734
Age 40	7.78	0.43	6.90	0.25	5.82	0.16	6.86	0.41	0.101	** < 0.001**	0.139	**0.001**	0.930	**0.027**

Statistically significant (*p* < 0.05) results are marked with bold font

#### Organised sports

At the baseline, the active and decreasingly active trajectories showed significantly higher membership levels in organised sports clubs compared with the other two trajectories (*p* < 0.01). From age 13 to 30 years, all trajectories showed a decrease in membership. However, the level of membership in sports clubs increased in the increasingly active trajectory from 0.67 at age 13 years to 0.76 at age 14 years and then decreased as for the other trajectory classes. From age 30 to 40 years, the mean level of membership in sports clubs increased among all four trajectories.

The active and low active trajectories differed significantly at all measurement points (*p* < 0.05). The decreasingly and increasingly active trajectories differed significantly (*p* < 0.05) at ages 13 and 14 years, when the decreasingly active trajectory showed higher participation levels. These trajectories did not differ at ages 15 to 18 years, but at age 23 years, the level of membership in sports clubs decreased more in the decreasingly active trajectory, which made the level in the increasingly active trajectory significantly higher (*p* < 0.01). At age 40 years, the membership level in organised sports was significantly higher in the active and increasingly active trajectories compared with the low active trajectory (*p* < 0.05).

Those inthe increasingly active trajectory reported the highest mean age at first participation in organised sports. However, the number of times reported being a member was not significantly lower than the active and the decreasingly active trajectories, whose mean age when becoming a member was younger.

#### Diversity in leisure-time activities

At age 15 years, the mean level of self-reported diversity in leisure-time activities was significantly higher in the active and decreasingly active trajectories than in the low active and increasingly active trajectories (*p* < 0.05). At age 30 years, the active and increasingly active trajectories exhibited greater diversity than the low active and decreasingly active trajectories. From the age of 30 to 40 years, diversity decreased in all trajectories, and the mean diversity in leisure-time activities did not differ significantly between the active, decreasingly active, and increasingly active trajectories. However, all three differed significantly from the low active trajectory.

#### Outdoor recreation

People in the low active trajectory reported significantly lower (*p* < 0.05) mean levels of outdoor recreation than those following the active trajectory at all measurement points, except during summer at age 13 years. The mean level also differed significantly between the low active and decreasingly active trajectory at most of the measurement points during adolescence. However, from age 23 years, the mean levels did not differ between these two trajectories except for outdoor recreation during the winter at age 40 years, when the low active class reported significantly lower mean levels. The low active trajectory class reported significantly lower outdoor recreation levels than those in the increasingly active trajectory class except at age 13 and in winter at age 15 years.

At age 23, we also found significantly lower (*p* < 0.05) mean levels of outdoor recreation during both summer and winter for the decreasingly active trajectory than for the active trajectory, and during winter compared to the increasingly active trajectory. At age 30, both the active and the increasingly active trajectories showed significantly higher (*p* < 0.05) mean levels of outdoor recreation than the decreasingly active trajectory. At age 40 years, there was a significant (*p* < 0.05) lower mean level of outdoor recreation during winter in the decreasingly active trajectory compared with the active trajectory.

#### Active peers

The number of active friends decreased in all classes from age 13 to 23 years. The number of active friends was significantly higher (*p* < 0.01) at all three measurement points in the active, decreasingly active, and increasingly active trajectories than in the low active trajectory. At age 15 years, the difference was also significant (*p* < 0.05) between the active and increasingly active trajectories. At age 18 years, the active and the decreasingly active trajectories differed significantly (*p* < 0.01).

At ages 13 and 15 years, those in the active trajectory reported significantly higher (*p* < 0.001) mean level of best friend’s PA compared with the low active and increasingly active trajectories. At age 13 years, the reported level of best friend’s PA differed significantly (*p* < 0.05) between the low active and increasingly active trajectories compared with the decreasingly active trajectory. At age 15 years, best friend’s PA differed significantly (*p* < 0.05) between all trajectories. However, at age 23 years, the active and increasingly active trajectories reported the same mean best friend’s PA, which was significantly higher (*p* < 0.01) than that reported for the other two trajectories.

#### Related demographic variables

Mean BMI did not differ significantly between the four trajectories. Having children was more frequently reported in the low active trajectory at age 21 years, when this frequency differed significantly (*p* < 0.01) from those of the active and increasingly active trajectories. At age 23 years, the frequency of having children differed (*p* < 0.05) only between the low active and increasingly active trajectories. At age 40 years, the frequency of having children differed significantly (*p* < 0.05) between the decreasingly active and the low active and increasingly active trajectories. Income at age 23 years did not differ significantly between the four trajectories, although the mean income at age 30 years was significantly higher (*p* < 0.01) in the active trajectory than in the other trajectories. At age 40 years, the low active trajectory had a significantly lower (*p* < 0.05) mean income than the other trajectories.

## Discussion

In this study, we aimed to identify developmental patterns of LVPA in a Norwegian sample with a follow-up of 27 years. We identified four LVPA trajectory classes from early adolescence to middle adulthood; these results partly support the findings of two cohort studies from Finland [7, 33].

The largest trajectory identified was the low active trajectory, which included slightly more than half of the sample (54%). Previous studies have also found a proportionally larger trajectory class with a persistently low PA level [8, 33–35]. Those following the low active trajectory reported the lowest mean LVPA level at all measurement points and showed considerable stability in their development. These results support the findings of a systematic review that showed that the low active trajectories appear to be more stable than more active trajectories [7] and a previous study showing that inactivity tracks better than activity [6].

The active trajectory appears at the other end of the spectrum and had the highest average level of LVPA at all measurement points, although it represented only 9% of the sample. A larger decreasingly active trajectory (25% of the sample) was identified at about the same high level of weekly LVPA as the active trajectory at age 13 years. From the age of 13 to 23 years, the weekly LVPA level decreased markedly in this trajectory. However, the LVPA level did not decline to the level of the low active trajectory, a finding that is consistent with previous research [7]. It is possible that high LVPA levels during adolescence may have contributed to a later decline in PA and prevented an earlier onset of lower activity. An increasingly active trajectory, representing 12% of the sample, was identified that exhibited a slightly different development from the other trajectory classes because the weekly LVPA increased from age 13 to 40 years.

Attention has focused on establishing habits when promoting lifelong engagement in PA. As indicated in the habit-formation hypothesis [6], repetition and routine are needed to form lasting habits. People in the active trajectory class are highly engaged in all activity domains from adolescence to adulthood. This consistency in engagement probably entails repeated PA behaviour over time, which increases the potential for establishing long-lasting PA habits.

The respondents following the decreasing trajectory may be labelled “early bloomers”. They had a high engagement in various PA domains in early adolescence, much like those in the active trajectory. However, their engagement and LVPA level declined from adolescence to young adulthood. The transition from adolescence to early adulthood reflects changes in the types of activities that are available and other critical life transitions such as moving away from home or starting higher education. As indicated in the carry-over value hypothesis, such changes can make continuing PA difficult if the possibility of engaging in the same type of activity as before is reduced [6]. It seems that people in the decreasingly active trajectory continue to engage in various activities during adolescence, which suggests some habitual behaviour but of lower intensity.

The findings of the current study also suggest that the people in the increasing trajectory were “late bloomers”, with a higher LVPA level during young adulthood and adulthood than in adolescence. However, the late onset does not seem to have hindered the positive development of a physically active lifestyle. The increasingly active trajectory was the only trajectory whose activity level continued to increase in young adulthood. The ability and readiness hypothesis suggests that experiences in PA and sports, such as organized sports, and the related basic skills, make it easier to continue with PA or to re-engage after a break. Thus, previous experience is valuable even though the type of activities and domains may differ at a later stage [6], for instance, PA at fitness centres or organised sports clubs for students.

### Activity domains in relation to trajectories

#### Membership in organised sports clubs

The active trajectory was characterised by higher participation in organised sports. However, at age 13 years, almost half the respondents in the low active trajectory also reported membership in organised sports, which suggests that membership alone does not necessarily guarantee higher levels of LVPA among adolescents. Similar results based on objectively measured PA have been reported in a youth sample from Finland [16]. There are several possible reasons why membership in organised sports did not ensure higher levels of LVPA for those in the low active trajectory in our study. One reason is the nature of the measurement used in this study, which did not include the frequency, duration, or intensity of PA related to the respondents’ engagement in sports clubs. The respondents may be members of an organised sports club but may participate seldom or have low engagement. Negative experiences (e.g., low motivation or mastery, conflict with coaches or peers) may also explain why those in the low active trajectory did not continue to participate in organised sports for extended periods, as indicated by the low average frequency of reported club membership. Therefore, their experiences with organised sports may have been too brief to make a lasting impact on their PA during the life course.

Respondents following the decreasingly active trajectory also reported high levels of membership in sports clubs, becoming a member of a sports club at an early age, and being a member at multiple measurement points. However, there did not seem to be a carry-over effect of these prior experiences, as shown by their decreasing activity level from age 13 to 23 years. This might be related to factors outside of sports clubs, such as injury, new interests and priorities, or different experiences with sports clubs or activities. Therefore, it is important to acknowledge that the same activity domain can contribute to both positive and negative experiences, which may influence the development of LVPA over time in different directions.

#### Active peers 

As supported by a previous study [19], peers can affect PA behaviour across, and in addition to, other domains. The peers’ activity levels may contribute to an increase or decrease in a person’s LVPA level. For example, the periods of adolescence and transition into young adulthood carry multiple opportunities to establish new relationships. We found an increase in the best friend’s activity level during the transition from adolescence to young adulthood among those in the increasingly active trajectory but a decrease among those in the decreasingly active trajectory. These findings emphasise the importance of peers to the development of LVPA and highlight how health promotion interventions should consider including peer relationships in initiatives to promote PA [19].

#### Diversity in leisure-time activities

Consistent with the ability and readiness hypothesis [6], we found that respondents in the trajectories exhibiting a stable engagement in a diversity of leisure-time activities either increased or maintained their relatively high LVPA level. By contrast, respondents in the decreasingly active trajectory showed a greater decrease in diversity in leisure-time activities from adolescence to young adulthood. Engagement in multiple sports and PAs during adolescence may provide an important base for developing motor skills and promoting long-lasting engagement in higher LVPA level later in life. This association has been shown in longitudinal studies [11, 12, 36]. Continuing to participate in several different sports or activities throughout adolescence and into adulthood may have contributed to the maintenance of a higher LVPA level, even though the number of activities the respondents participated in at age 40 years did not differ between the active, increasingly active, and decreasingly active trajectories.

#### Outdoor recreation

All trajectories showed relatively high levels of outdoor recreation during both summer and winter during adolescence. Engagement in outdoor recreation is closely linked to cultural characteristics in Norway, and it is common to spend leisure time outdoors, especially during weekends and holidays [12]. We found significant differences indicating that respondents in the low active trajectory engaged less in these activities than the other trajectories. However, our findings do not allow us to determine how outdoor recreation contributed to the LVPA level. By being accessible to all and not requiring membership or special equipment, outdoor recreation may contribute to LVPA throughout the life course more broadly than organised sports or PA at fitness centres. In our study, respondents following the low active trajectory seemed to be less active in outdoor recreation during winter compared with the other trajectories. Outdoor recreation in winter can be challenging in a Nordic country like Norway. Such seasonal changes may be important to consider when planning initiatives to promote activity, at least for outdoor recreation during winter among those in the low active trajectory.

### Demographic and socio-economic status across the LVPA trajectories

The active and decreasingly active trajectories were more prevalent among males than females, which is consistent with earlier reports [7, 16, 25]. In contrast to previous research on the relationship between BMI and PA development [23, 26, 27], our study did not find differences in these parameters between the four trajectories. At age 21 years, the low active trajectory was characterised by more respondents having children compared with the two trajectories with the highest LVPA level at that time. Previous research has found that having a child is negatively associated with overall PA [24], that having children increases the odds of belonging to the decreasingly active trajectory [8], and that, among women, having children has a strong negative effect on the number of sports practised [28]. At age 40 years, a large proportion of the respondents reported having children. In Norway, most organised sports clubs rely on parental engagement and voluntary work, which may explain the increase in sports club membership at age 40 years among the respondents in our study. Our findings of significant differences in income between the active and low active trajectories are consistent with those of studies analysed in a recent systematic review [7] and suggest that mean income is higher in the active trajectory.

### Limitations

This study has some limitations. Self-reporting might lead to over- or under-reporting [37]. We used a single item regarding frequency to measure LVPA, which may have oversimplified this phenomenon with many different dimensions, such as type of activity or duration. However, use of this single-item question has been shown to have acceptable reliability and validity [21, 38]. Further validation studies are needed [39], including validation across the life course. Adding a measure of moderate physical activity or sedentary behaviour could have enriched our analysis, however these types of measurements were not available in the data material.

We assessed participation in organised sports using a single item related to sport club membership. Membership does not necessarily indicate active participation, and having more items related to sports participation, such as frequency and level of activity, may have strengthened our analysis. This is also relevant to many of the other items related to activity domains. For example, to assess the diversity in leisure-time activities, we created a sum score based on more detailed information related to the types of activities and frequencies. Excluding this sum score and analysing specific details may have added valuable information about the degree of active participation in different activities across the four trajectories. However, for some activities, the number of respondents was too low, and we would not have been able to include the whole range of different activities in the analyses.

The analyses did not control for any variables, and the differences between the classes on the auxiliary variables may reflect an effect of confounders. We did not assess the strength of the associations or compare them with each other but examined only the different characteristics of the four latent trajectory classes grouped according to LVPA. Lastly, to summarise data, a reduction is sometimes needed. In group-based models, one reduces by approximation and by grouping, and comparing individuals who are not entirely homogenous [30]. Therefore, it is essential to recognise that trajectory group membership is not definite because the LCGA gives only the probability of following a trajectory. Further, our results are not necessarily generalisable to other populations as the studied sample represents a population from western Norway. However, the baseline mean of LVPA at age 13 in this sample (collected in October/November 1990) was almost identical to that of the nationally representative HBSC study sample of 13-year-olds (*n* = 1616) collected in November 1989, suggesting that the study sample from western Norway at baseline was representative of Norwegian youth in the relevant age group [12]. The main strength of the present study is its longitudinal design and long-time follow-up with many measurement points, relatively large sample size, and comprehensive use of self-reported measures of LVPA and engagement in different activity domains. In addition, using finite mixture modelling of longitudinal data provides new information about the complexity of PA behaviour over that provided by population-based mean levels.

## Conclusions

Four life-course trajectories of LVPA were identified in this 27-year longitudinal study in Norway. Primarily, the findings suggest heterogeneity in the development of LVPA across different life periods. Secondly, the largest trajectory group included more than half of the respondents and was characterised by a low LVPA level and low engagement in the four activity domains, calling for increased recruitment into different activity domains for children and adolescents at high risk of falling into this trajectory. Thirdly, engagement in organised sports in adolescence does not seem to have a sustainable carry-over effect on the level of LVPA later in life. For organised sports clubs to contribute to life-long PA, there needs to be more awareness on drop-out and retention. Lastly, those belonging to the more active trajectories reported having more active peers, indicating that the social impact of friends influences the activity level. In the development of PA, changing social surroundings during life may assist or hinder health enhancing engagement in LVPA. This and the heterogeneity in LVPA development from adolescence to adulthood highlights the need for targeted and age group specific health promotion initiatives.

## Supplementary Information

**Additional file 1.** Information about the recruitment and representativeness of the sample, consent, data collection and how missing data was handled.

**Additional file 2.** Table showing the different types of leisure-time activities that were included in the questionnaire in 1992, 2000 and 2017. These data were used to make the measure of diversity in leisure-time activities.

**Additional file 3.** The plots for the one- to seven-class solutions for the LCGA are shown. The plots show the sample mean of leisure-time vigorous physical activity (times per week) from age 13 to age 40 (n=1103).

**Additional file 4.** Completed checklist for guidelines for reporting on latent trajectory studies (GRoLTS).

**Additional file 5.** Completed STROBE checklist.

## Data Availability

The data and materials used for the current study are available from the corresponding author upon reasonable request.

## Abbreviations

AIC: Akaike information criterion
BCH: Block–Croon–Hagenaars
BIC: Bayesian information criterion
BLRT: Bootstrap likelihood ratio test
BMI: Body mass index
CFI: Comparative fit index
FIML: Full information maximum likelihood
GRoLTS: Guidelines for Reporting on Latent Trajectory Studies
HBSC: The Health Behaviour in School-aged Children (HBSC) Study, WHO Collaborative Cross-national Study
LCGA: Latent class growth analysis
LVPA: Leisure-time vigorous physical activity
MAR: Missing at random
MLR: Maximum likelihood estimator with robust standard error
PA: Physical activity
RMSEA: Root mean square error of approximation
VLMR: Vuong–Lo–Mendell–Rubin test
