# Supplementary material for: Leisure-time physical activity trajectories from adolescence to adulthood in relation to several activity domains: a 27-year longitudinal study
Source: Int J Behav Nutr Phys Act. 2023 Mar 9;20:27. doi: 10.1186/s12966-023-01430-4 (PMC9996998; doi:10.1186/s12966-023-01430-4)
Supplement: Supplementary file 1 — Additional file 1. Information about the recruitment and representativeness of the sample, consent, data collection and how missing data was handled. [file 12966_2023_1430_MOESM1_ESM.pdf]

Additional information about the sample

The NLHB study follows the same participants from age 13 in 1990 to age 40 in 2017, in all ten times. See Table 1 below for the number of participants and age at each data collection. The sample was drawn from 22 randomly selected schools (54 classes), picked systematically from an alphabetical list of all schools in the region. To allow close contact with the participating students in the important early phase of the project the sample was limited geographically to the county of Hordaland on the west coast of Norway. At the first data collection in 1990, nine hundred and twenty-four students participated. This was 77% of the 1195 students who were initially invited to take part. Excluded from this sample were those whose parents did not give their consent for participating or who failed to return the consent form (n=222); those who did not wish to participate (n=46); and those who provided unusable responses (n=3).

Any new student in the included schools in 1991 and 1992 were invited to participate, increasing the total number of students invited by 47, resulting in a total sample of 1242. Twenty nine of these did not participated in 1990,1991 or 1992, but participated at least once during the data collections between 1993 and 2017.

Table 1. Measurement years, age and total number of participants per measurement year.

	1990	1991	1992	1993	1995	1996	1998	2000	2007	2017
Age	13	14	15	16	18	19	21	23	30	40
N	924	958	963	789	779	643	634	627	536	455

For the present data analysis, participants with at least one measurement of LVPA over the ten measurement points were included. This reduced the included sample to 1103 participants. Missing data were assumed to be missing at random (MAR) and addressed using full information maximum likelihood estimation (FIML).
