# Supplementary material for: Leisure-time physical activity trajectories from adolescence to adulthood in relation to several activity domains: a 27-year longitudinal study
Source: Int J Behav Nutr Phys Act. 2023 Mar 9;20:27. doi: 10.1186/s12966-023-01430-4 (PMC9996998; doi:10.1186/s12966-023-01430-4)
Supplement: Supplementary file 2 — Additional file 2. Table showing the different types of leisure-time activities that were included in the questionnaire in 1992, 2000 and 2017. These data were used to make the measure of diversity in leisure-time activities. [file 12966_2023_1430_MOESM2_ESM.pdf]

Type of leisure-time activity	*Included in questionnaire* 1992	2000	2017
Walking/hiking	x	x	x
Walking fast > 10 minutes	x	x	x
Hard work (house, garden, etc.)	x	x	x
Cycling to school/work/etc. > 10 minutes	x	x	x
Jogging alone	x	x	x
Jogging with others	x	x	x
Aerobics	x	x	x
Dancing	x	x	x
Weight or power lifting	x	x	x
Martial arts	x	x	
Body building	x	x	
Judo, karate	x	x	
Soccer	x	x	x
Basket, volley, handball	x	x	x
Diving	x	x	x
Cycling	x	x	x
Gymnastics	x	x	
Track running	x	x	
Track & field	x	x	
Table tennis	x	x	
Badminton, tennis, squash	x	x	x
Horse riding	x	x	
Archery, golf, bowling	x	x	
Rowing, canoeing	x	x	
Orienteering (in summer)	x		
Windsurfing, sailing (in summer)	x	x	x
Diving (in summer)	x	x	
Jet ski (in summer)	x	x	
Hang-gliding, air sport (in summer)	x	x	
Skating, ice hockey, bandy (in winter)	x	x	x
Downhill, ski jumping (in winter)	x	x	x
Cross-country skiing, biathlon (in winter)	x	x	x
Fitness centre		x	x
Other	x	x	x
