# Supplementary material for: Leisure-time physical activity trajectories from adolescence to adulthood in relation to several activity domains: a 27-year longitudinal study
Source: Int J Behav Nutr Phys Act. 2023 Mar 9;20:27. doi: 10.1186/s12966-023-01430-4 (PMC9996998; doi:10.1186/s12966-023-01430-4)

Plot of the one-, two-, three-, five-, six-, and seven-class solution for the Latent class growth analysis
Sample mean of leisure-time vigorous physical activity, times per week

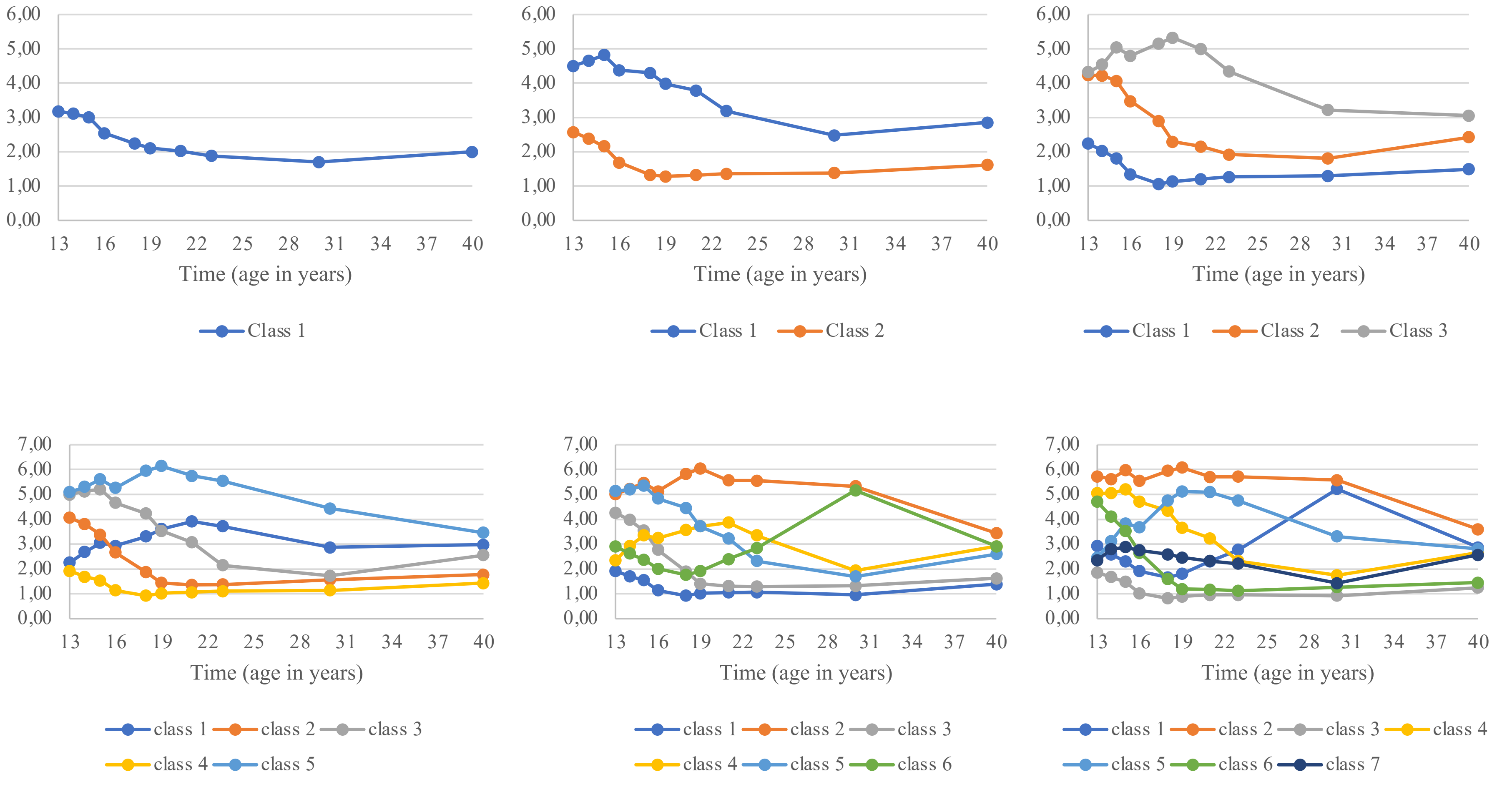

Supplement: Supplementary file 3 — Additional file 3. The plots for the one- to seven-class solutions for the LCGA are shown. The plots show the sample mean of leisure-time vigorous physical activity (times per week) from age 13 to age 40 (n=1103). [file 12966_2023_1430_MOESM3_ESM.pdf]
