# Supplementary material for: Leisure-time physical activity trajectories from adolescence to adulthood in relation to several activity domains: a 27-year longitudinal study
Source: Int J Behav Nutr Phys Act. 2023 Mar 9;20:27. doi: 10.1186/s12966-023-01430-4 (PMC9996998; doi:10.1186/s12966-023-01430-4)
Supplement: Supplementary file 4 — Additional file 4. Completed checklist for guidelines for reporting on latent trajectory studies (GRoLTS). [file 12966_2023_1430_MOESM4_ESM.pdf]

Additional file 1
Filled out checklist for guidelines for reporting on latent trajectory studies (GRoLTS)*.

	Checklist Item	Reported?
1.	Is the metric of time used in the statistical model reported?	Yes
2.	Is information presented about the mean and variance of time within a wave?	No
3a.	Is the missing data mechanism reported?	Yes
3b.	Is a description provided of what variables are related to attrition/missing data?	No
3c.	Is a description provided of how missing data in the analyses were dealt with?	Yes
4.	Is information about the distribution of the observed variables included?	Yes
5.	Is the software mentioned?	Yes
6a.	Are alternative specifications of within-class heterogeneity considered (e.g., LGCA vs. LGMM) and clearly documented? If not, was sufficient justification provided as to eliminate certain specifications from consideration?	No
6b.	Are alternative specifications of the between-class differences in variance-covariance matrix structure considered and clearly documented? If not, was sufficient justification provided as to eliminate certain specifications from consideration?	No
7.	Are alternative shape/functional forms of the trajectories described?	Yes
8.	If covariates have been used, can analyses still be replicated?	Not used
9.	Is information reported about the number of random start values and final iterations included?	Yes
10.	Are the model comparison (and selection) tools described from a statistical perspective?	Yes
11.	Are the total number of fitted models reported, including a one-class solution?	Yes
12.	Are the number of cases per class reported for each model (absolute sample size, or proportion)?	Yes
13.	If classification of cases in a trajectory is the goal, is entropy reported?	Yes
14a.	Is a plot included with the estimated mean trajectories of the final solution?	Yes
14b.	Are plots included with the estimated mean trajectories for each model?	Yes, sample mean
14c.	Is a plot included of the combination of estimated means of the final model and the observed individual trajectories split out for each latent class?	No
15.	Are characteristics of the final class solution numerically described (i.e., means, SD/SE, n, CI, etc.)?	No
16.	Are the syntax files available (either in the appendix, supplementary materials, or from the authors)?	Yes, from the authors

*Rens van de Schoot, Marit Sijbrandij, Sonja D. Winter, Sarah Depaoli & Jeroen K. Vermunt (2017) The GRoLTS-Checklist: Guidelines for Reporting on Latent Trajectory Studies, Structural Equation Modeling: A Multidisciplinary Journal, 24:3, 451-467, DOI: 10.1080/10705511.2016.1247646
